# Contribution of SLC7A1 genetic variant to hypertension, the TAMRISK study

**DOI:** 10.1186/1471-2350-14-69

**Published:** 2013-07-10

**Authors:** Kirsi Määttä, Tarja Kunnas, Seppo T Nikkari

**Affiliations:** 1 Department of Medical Biochemistry, University of Tampere Medical School and Fimlab laboratories, Tampere 33014, Finland

## Abstract

**Background:**

The rs41318021 polymorphism in the SLC7A1 gene affects endothelial NO production through changes in L-arginine transport. This variation could thus hypothetically cause dysfunction of endothelium and lead to hypertension. The association of rs41318021 with hypertension was therefore studied in a Finnish cohort.

**Methods:**

A total of 412 hypertensive cases and 771 non-hypertensive controls from a Finnish 50-year-old cohort were included in this study. The data was collected from the Tampere adult population cardiovascular risk study (TAMRISK). DNA was extracted from buccal swabs and amplified using PCR. A subpopulation of men and women who had available follow-up data of blood pressure measurements at the age of 35-, 40-, 45- and 50 years was also analyzed.

**Results:**

There was no difference between the variant frequencies of the hypertension group and normotensive group at the age of 50 years (p = 0.209). However, repeated measures analysis from the 15-year follow-up showed that subjects having gene variants CT or TT had slightly higher diastolic blood pressure than subjects having genotype CC (p = 0.047). By post-hoc analysis, this was most pronounced at the age of 35 years (p = 0.044).

**Conclusion:**

The rs41318021 polymorphism in the SLC7A1 gene was not associated with essential hypertension in 50-year-old subjects. However, a borderline effect of this variation upon diastolic blood pressure was seen in these same subjects in a 15-year follow-up from a 35-year-old cohort to 50 years of age.

## Background

Hypertension is a disorder caused by a complex combination of genetic and lifestyle risk factors. Hypertension raises the risk of incident cardiovascular disease to two-or three-fold [1]. The major contributors to hypertension in Western countries are overweight, physical inactivity, high salt intake and low potassium intake [2]. The genetic component in hypertension is estimated as 30 – 50% of the total impact [3]. Candidate-gene and genome-wide association studies (GWAS), have previously identified numerous genetic loci that are associated with blood pressure, but collectively these explain only a few percent of the heritability for hypertension [4,5].

The role of vascular endothelium is significant in the regulation of blood pressure. Endothelial cells synthesize vasodilatory factors like nitric oxide (NO). Arginine is the rate limiting substrate for endothelial nitric oxide synthase (eNOS), which acts as a catalyst in NO production [6-8]. SLC7A1 (previously CAT-1), located in chromosome 13q12-q14, encodes a cationic amino acid transporter for arginine and lysine uptake in mammalian cells [9,10]. Functional variation of SLC7A1 gene alters the expression of SLC7A1, which may result in changes of NO production and endothelial function [10]. Therefore, changes in SLC7A1 gene could cause dysfunction of endothelium and lead to hypertension.

To our knowledge, there is only one previous study that has addressed the association of rs41318021 with hypertension in an Australian population [10]. We wanted to assess the role of this variant in a Finnish population, by analyzing cohorts from the Tampere adult population cardiovascular risk study (TAMRISK).

## Methods

### Subjects

The data for the TAMRISK study was collected from periodic health examinations (PHE) done for 50-year-old men and women living in Tampere, Finland. TAMRISK data includes information of risk factors for hypertension: blood pressure, weight, family history of cardiovascular diseases, lipid values and smoking, diabetes and exercise habits [11]. Buccal swabs for DNA extraction and a permissions form to use PHE data were collected by mail separately of the physical examination. The DNA samples were collected during years 2006–2010. Informed consent was obtained from all participants. The Ethics Committees of the Tampere University Hospital and the City of Tampere approved the study.

Cases (n = 412) in this study were the subjects who had hypertension at the age of 50 years (as diagnosed by a physician) and for each case, at least one normotensive control (n = 771) with the same sex and similar smoking habits, were chosen from a PHE cohort (n = 6000). Smoking status was evaluated based on self-reporting. Finally, we selected a subpopulation of men and women who had available data of blood pressure measurements at the age of 35-, 40-, 45- and 50 years.

### Baseline measurements

The basic evaluation in 1988–91 included an interview by a public health nurse. The interview was conducted using a structured questionnaire about health and health-related behaviour, including questions about current and previous diseases. Information on current and previous diseases was based on self-report of diagnosis by a physician, including history of coronary artery disease, myocardial infarction and diabetes. Family history of hypertension in a close relative was also asked in the questionnaire. The frequency of physical exercise comprised both leisure and commute related activity. Physical examination included a single blood pressure (BP) measurement (mm of mercury) using a calibrated mercury sphygmomanometer. Serum total cholesterol (mmoles/litre), glucose (mmoles/litre) and haemoglobin (grams/litre) were measured after an overnight fast by standard techniques. Height (cm) and weight (kg) were recorded from which the body mass index was calculated.

### Genotyping

DNA was extracted from buccal swabs using a commercial kit (Qiagen Inc., Valencia, Calif., USA). Genotyping was performed using allele specific primers: SLC7A (047) 5′-AGT TGT CTG GAG GTG ACC-3′ and SLC7A (050 T) 5′-GCAAGTGACGCACAGCCT-3′ or SLC7A (049C) 5′-GCAAGTGAGGCACAGCCC-3′ as described by Yang et al. (2007) [10]. Two parallel PCRs were performed for each sample. Amplification was done in 94°C for 15 minutes followed by 32 cycles of 94°C, 55°C and 72°C for 30 s each. The final extension was at 72°C for 5 min. The PCR products were resolved in 2% agarose gels.

### Statistical analysis

Analysis of variance (ANOVA) for continuous variables and Chi-square test for categorical variables were applied for the comparison of cases and controls at the age of 50 years. If the distribution was skewed, the analysis was performed using transformed values to approximately normalize the distribution. To assess the effect of risk factors for hypertension, we used binary logistic (forwald wald) regression analysis. The analysis of variance for repeated measures was used to assess the differences in mean blood pressures between genotypes at the age of 35-, 40-, 45- and 50 years. This follow-up data was available from 775 participants.

P values less than 0.05 were considered significant. Analyses were carried out using SPSS 16.0 for Windows (SPSS Inc., Chicago, Illinois, USA).

## Results

Clinical characteristics of the cases (412) and controls (771) at the age of 50 years are presented in Table 1. The case group of hypertensive subjects was compared to healthy controls. Cases had higher body mass index (BMI), haemoglobin (Hb), triglycerides, fasting glucose, systolic and diastolic blood pressure, and lower HDL cholesterol compared to controls. Cases exercised more than controls and had higher frequency of diabetes, myocardial infarction and family history of hypertension.

**Table 1:** Clinical characteristics of study population at the age of 50 years

	**Cases (n = 412)**	**Controls (n = 771)**	**P**
Age (years)	50 ± 0	50 ± 0	
BMI (kg/m^2^)	28.6 ± 5.1	25.4 ± 3.6	<0.001
Haemoglobin (g/l)	147.2 ± 13.2	143.90 ± 13.6	<0.001
Cholesterol (mmol/l)	5.40 ± 0.97	5.40 ± 0.92	0.965
HDL cholesterol (mmol/l)	1.56 ± 0.45	1.67 ± 0.44	<0.001
LDL cholesterol (mmol/l)	3.17 ± 0.87	3.21 ± 0.84	0.512
Triglycerides (mmol/l)	1.55 ± 1.20	1.17 ± 0.68	<0.001
Glucose (mmol/l)	5.14 ± 1.20	4.84 ± 0.55	<0.000
Systolic blood pressure (mm Hg)	142.93 ± 16.17	129.75 ± 14.66	<0.000
Diastolic blood pressure (mm Hg)	92.92 ± 8.95	84.44 ± 9.40	<0.000
Exercise (at least twice a week) %	33.8	28.0	0.048
Diabetes %	11.8	0	<0.000
Myocardial infarction %	2.5	0	<0.000
Family history of hypertension %	71.3	42.2	<0.000
Current smoking %	19	19	
Gender (male) %	58.4	56.8	0.319

Data are presented as mean ± SD.

In the whole study population the frequencies of the rs41318021 variants for SLC7A1 were 0.70 for CC (n = 828), 0.27 for CT (n = 319) and 0.03 (n = 36) for TT. There was no difference between the variant frequencies of the hypertension group and normotensive group (p = 0.209). In the hypertension group the frequencies were 0.69 for CC, 0.29 for CT and 0.02 for TT. In the control group these proportions were 0.71, 0.26 and 0.03, respectively.

To find the explainable factors for hypertension in the 50-year-old cohort we analyzed the data using logistic regression analysis. When SLC7A1 genotype, BMI, Hb, HDL cholesterol, triglycerides, glucose, and family history of hypertension were included in stepwise analysis (forward wald), BMI, (p < 0.001, OR: 1.19, 95% CI 1.14-1.24), glucose (p = 0.009, OR: 1.37, 95% CI: 1.08-1.75) and family history of hypertension (p < 0.001, OR: 3.28, 95% CI: 2.35-4.56) were associated with hypertension.

**Figure 1:**
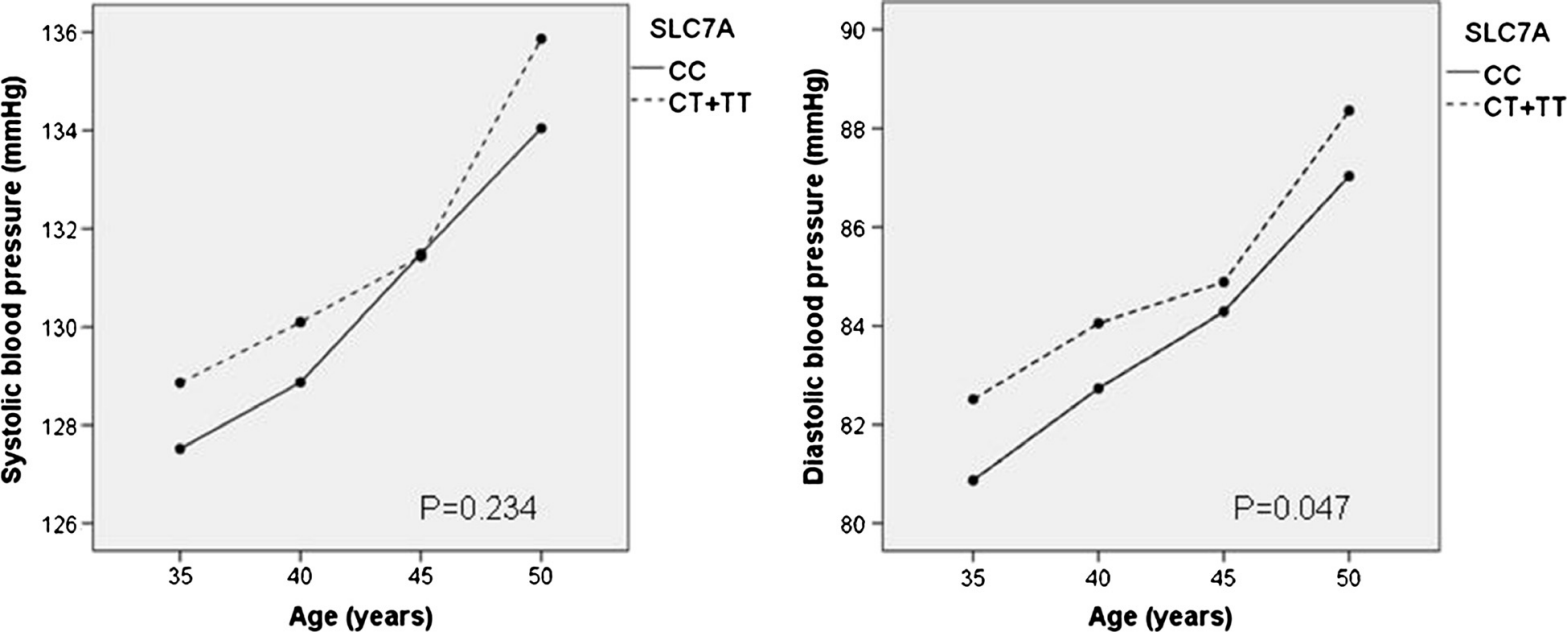
Means of systolic and diastolic blood pressures in follow-up at the age of 35-, 40-, 45- and 50 years, according to SLC7A1 gene variants.

Finally we selected a subpopulation of men and women in the present study who had available data of blood pressure measurements at the age of 35-, 40-, 45- and 50 years. This follow-up data was available from 775 participants, with subjects from both the hypertension and control groups. Subjects with CT and TT genotypes were combined, because there were only 23 subjects in the TT group. Repeated measures analysis showed that subjects having gene variants CT or TT had slightly higher diastolic blood pressure than subjects having genotype CC (p = 0.047, Figure 1). The difference between genotypes in diastolic blood pressure was 1.4 mmHg at its highest at the age of 35 years. The trend was similar for systolic blood pressure, but not statistically significant (p = 0.234). By one way ANOVA, the only statistical difference for diastolic and systolic blood pressure between the genotypes was for diastolic blood pressure at the age of 35 years (p = 0.044).

When covariates that emerged as being significant in the logistic regression at the age of 50 years (BMI and glucose) were added to the repeated measures model, the results remained the same in the follow-up. Subjects having gene variants CT or TT had higher diastolic blood pressure than subjects having genotype CC (p = 0.021). Again, the trend for systolic blood pressure was not statistically significant (p = 0.241).

## Discussion

Gene variation rs41318021 in the human SLC7A1, which encodes the principal arginine transporter, has previously been proposed to associate with a genetic predisposition to essential hypertension [10,12]. Our study subjects were from Finland, where it is estimated that over 48% of 25–64 year old adults have mean systolic pressure of 140 mmHg or above, which is among the highest in Europe [2]. The association of rs41318021 with hypertension was therefore studied in this Finnish cohort.

Environmental and genetic changes in L-arginine transport are known to influence NO production significantly. Endothelial NO formation is reduced in subjects with essential hypertension, when compared to normotensive subjects [12]. Synthesis of NO requires substrate L-arginine [13]. Vasodilatation may be affected by limitation of substrate (L-arginine) for the synthesis of NO. The actual effect of decreased availability of L-arginine on impaired endothelial function is still controversy. Increasing age is associated with declining endothelial function [14], which might explain some of the differences between contradictory results on L-arginine availability and hypertension [15,16].

L-arginine transporter SLC7A1 (previously CAT-1) has an important role in L-arginine availability [8]. Yang and Kaye [17] were able to show differences in gene expression between alleles of the SLC7A1 gene (rs41318021). Using reporter gene constructs they showed that minor allele T in rs41318021 is associated with lower gene expression compared with major allele C. They also found that there was a difference in allele frequency between hypertensive and normotensive subjects in an Australian population. In hypertensive subjects the frequency of the T allele was 13.3%, compared with 7.6% in the normotensive subjects (P < 0.001) [10,17].

The frequencies of the SLC7A1 gene variants in our study population were similar to those reported previously [10]. In contrast to the earlier study [10], polymorphism rs41318021 in the SLC7A1 was not associated with essential hypertension in 50-year-old subjects. On the other hand, a borderline effect of this variation was seen in these same subjects from the age of 35 years. This is in line with the conclusion that the effect of a single gene polymorphism is seen more likely in a younger population [5]. The observed effect of a single gene variation on blood pressure in GWAS studies is low: +1.16 mmHg at the highest [5], as also seen in the present study.

The background of hypertension consists of many genes and moreover of gene-environment interactions. Ageing is known to result in untoward increases in body mass index in the TAMRISK study cohorts [11]. Thus, genetic effects are more likely to be seen at younger ages. With aging, other factors, e.g. overweight, may be somewhat more relevant to development of hypertension.

## Conclusions

In conclusion, the effect of gene variation rs41318021 in the human SLC7A1 on blood pressure was of borderline significance at the age of 35 years, and masked by environmental factors by the age of 50 years in the TAMRISK study.

### Consent

Written informed consent was obtained from all study subjects for the publication of this report.

## Competing interests

The authors declare that they have no competing interests.

## Authors’ contributions

KM contributed to the analysis and interpretation of the data and drafting the manuscript. TK and STN contributed to conception and design of this study, drafting the manuscript and revising the article critically for important intellectual content. All authors read and approved the final manuscript.

## Authors’ information

Tarja Kunnas and Seppo T Nikkari are senior authors.

## Pre-publication history

The pre-publication history for this paper can be accessed here:

http://www.biomedcentral.com/1471-2350/14/69/prepub
